# ASME-Based Structural Assessment of Head–Shell Junctions in Pressurized Railway Tank Wagons

**DOI:** 10.3390/ma19061125

**Published:** 2026-03-13

**Authors:** Costin Nicolae Ilincă, Rami Doukeh, Ibrahim Naim Ramadan, Adrian Neacsa, Alin Diniță, Eugen Victor Laudacescu, Marius Gabriel Petrescu, Bogdan Ilie, Andrei Cosmin Sîrbu

**Affiliations:** Mechanical Engineering Department, Petroleum-Gas University of Ploiesti, 100680 Ploiesti, Romania; icostin@upg-ploiesti.ro (C.N.I.); adnea@upg-ploiesti.ro (A.N.); leugen@upg-ploiesti.ro (E.V.L.); andrei-cosmin@upg-ploiesti.ro (A.C.S.)

**Keywords:** stress linearization, ASME Section VIII Division 2, finite element analysis (FEA), geometric discontinuity

## Abstract

This study presents an ASME-based structural assessment of the head–shell junction in a 60 m^3^ pressurized railway tank wagon subjected to an internal pressure of 0.45 MPa, combining classical shell theory with finite element analysis (FEA) in accordance with ASME Section VIII Division 2 stress categorization and linearization procedures. An analytical model based on the moment theory of shells of revolution was developed to describe displacement and rotation compatibility at the ellipsoidal head–cylindrical shell junction, allowing for the determination of contour interaction loads governing membrane–bending coupling in the discontinuity region. The calculated contour loads (Q_0_ = 795 N/mm, M_0_ = 13,350 N·mm/mm) indicate localized membrane–bending interactions caused by geometric discontinuity. Finite element simulations using axisymmetric (2D) and full 3D models were evaluated through the ASME VIII-2 stress linearization procedure, enabling comparison between analytical predictions and numerical results. The maximum equivalent stress according to the Coulomb–Tresca criterion reached 115 MPa (2D) and 117 MPa (3D), with less than 2% deviation, confirming the adequacy of the axisymmetric model. Stress linearization shows that the maximum combined primary membrane and bending stress (109.5 MPa) remains well below the ASME allowable limit of 308 MPa, while the discontinuity influence zone extends approximately 120–150 mm from the junction. The results confirm compliance with ASME VIII Division 2 requirements and demonstrate that the combined analytical–numerical approach provides a reliable method for evaluating stress concentration effects in railway tank wagons.

## 1. Introduction

Pressure vessels are important structural components in petrochemical, energy, and transportation systems, where their integrity is directly linked to operational safety and risk mitigation [1,2]. Due to the high mechanical energy stored under internal pressure, even localized defects may initiate cracking or leakage below nominal test pressure levels [3]. In railway tank wagons, vessels operate under internal pressure while simultaneously being subjected to transportation-induced loads, making structural reliability a primary safety requirement [4].

Of particular concern are geometric discontinuities, such as head–shell junctions, where abrupt changes in curvature and stiffness generate localized membrane–bending interactions and stress concentration effects. Corrosion defects or material imperfections in these regions may further amplify stress intensities and compromise structural integrity [2]. Consequently, accurate stress evaluation and discontinuity assessment are essential to prevent premature failure and ensure safe long-term operation.

The design and integrity verification of such pressurized structures are governed by internationally recognized standards, notably the ASME Boiler and Pressure Vessel Code, Section VIII Division 2 [5] and AS1210 [6]. While both standards aim to ensure safe and functional design, they provide different methodological frameworks for stress categorization, acceptance criteria, and defect assessment. In the context of head–shell junctions, ASME VIII Division 2 offers a structured approach based on stress linearization and classification, enabling a code-compliant evaluation of local stress concentrations.

Geometric discontinuities in pressure vessels, particularly at the head–shell junction, generate stress states that deviate from the ideal membrane condition due to curvature transition and displacement compatibility effects. In internally pressurized ellipsoidal heads, circumferential compressive stresses develop in the knuckle region and may induce localized bending and instability phenomena [7]. The resulting stress amplification is strongly influenced by geometric ratios (D/t, D/2 h) and fabrication imperfections such as weld bulging or thickness transitions. Similar mechanisms are observed at circumferential welds, where stiffness mismatch produces additional bending stresses [8]. Therefore, the head–shell junction must be treated as a structural discontinuity zone requiring detailed analytical or numerical assessment.

In this context, advanced numerical approaches are required to accurately quantify the resulting membrane–bending interaction and peak stress amplification in such discontinuity regions.

Finite Element Analysis (FEA) is widely employed in the literature to evaluate stress concentrations in pressure vessels, particularly at structural discontinuities such as nozzle–shell and head–shell junctions. Comparative investigations report good agreement between analytical solutions and FEA predictions, typically within 2–7%, confirming the reliability of numerical modelling for complex geometries [9]. Studies conducted in accordance with ASME Section VIII Division 2 consistently identify these junction regions as risky stress concentration zones, where membrane–bending interaction governs the local stress state [10]. Stress linearization along Stress Classification Lines (SCLs) enables separation of membrane and bending components for direct code verification, with deviations between analytical and numerical approaches often remaining within approximately 2% [11].

Beyond elastic verification, Fitness-for-Service (FFS) methodologies based on Failure Assessment Diagrams (FADs), as prescribed in ASME Sections III and XI, highlight the important role of stress categorization and reference stress evaluation in defect acceptability [12]. For instance, variations in stress classification significantly influence allowable crack dimensions under different service levels. Similarly, ASME fatigue provisions in Sections III and VIII are based on crack initiation rather than failure, meaning that allowable cycle limits depend strongly on accurate membrane–bending stress decomposition [13]. These considerations underline the importance of proper stress categorization in discontinuity regions such as head–shell junctions.

FEA studies on ASME-designed LPG vessels (10 m^3^ capacity) further show that maximum stresses typically occur at geometric discontinuities such as manways and junction interfaces, while remaining below the material ultimate strength (*S_u_* = 485 MPa). Increasing shell thickness reduces displacement and linearly improves the factor of safety, highlighting the sensitivity of local stress behaviour to geometric parameters [14].

Despite its extensive application, stress linearization procedures adopted in ASME VIII Division 2 retain a classical shell-theory basis. Recent investigations indicate that full tensor linearization in 3D solid models may violate surface boundary conditions, suggesting inherent limitations in the traditional membrane–bending stress decomposition framework [15].

Notwithstanding the extensive literature on pressure vessel discontinuities and finite element assessment, limited attention has been devoted to the integrated analytical–numerical evaluation of head–shell junctions in railway tank wagons within the framework of ASME Section VIII Division 2. In particular, a rigorous quantitative comparison between classical shell-theory-based predictions and stress-linearized finite element results for this specific configuration remains insufficiently documented. Moreover, the spatial extent and magnitude of membrane–bending interaction effects in such discontinuity zones have not been consistently assessed in a code-compliant manner.

While analytical solutions and finite element studies for head–shell junctions exist in the pressure vessel literature, the present work introduces several aspects that are less documented for railway tank wagon structures:

Compatibility-based analytical formulation of the head–shell junction, explicitly determining the contour interaction loads $Q_{0}$ and $M_{0}$ resulting from the displacement incompatibility between the cylindrical shell and the ellipsoidal head.

Direct quantitative comparison between classical shell theory predictions and stress-linearized finite element results obtained in accordance with ASME Section VIII Division 2 procedures.

Identification of the spatial extent of the discontinuity influence zone (approximately 120–150 mm from the junction contour) through both analytical and numerical approaches.

Application of ASME Div. 2 stress categorization and verification procedures to a railway tank wagon geometry, which differs from conventional stationary pressure vessels due to geometric proportions and transport-related design constraints.

Accordingly, the present study aims to bridge this gap by combining analytical shell theory with finite element modelling and ASME-based stress categorization procedures to quantify stress amplification at the head–shell junction and to verify structural integrity against the hierarchical acceptance limits prescribed by ASME VIII Division 2.

In addition to the structural integrity assessment itself, the present study establishes a direct correlation between analytical shell-theory predictions and stresses obtained from finite element analysis interpreted according to ASME Section VIII Division 2 stress linearization procedures. While analytical formulations and numerical simulations are frequently reported separately in the literature for pressure vessel discontinuities, their direct comparison within a code-consistent stress categorization framework is less commonly addressed [16]. The analytical formulation developed in this work explicitly introduces displacement and rotation compatibility conditions at the head–shell junction (Equations (12)–(19)), which allow the contour interaction loads to be determined in a physically transparent manner. This formulation enables a consistent comparison between the analytical stress solution and the membrane and bending stress components obtained from finite element stress linearization, providing a clearer interpretation of stress redistribution in the discontinuity region.

The cylindrical shell–head junction is a structural discontinuity zone where local bending effects and stress concentration may become significant under internal pressure [16,17,18]. Recent studies confirm that analytical shell theory and edge-effect formulations remain useful for such problems, especially when correlated with finite element analysis [17,19]. In this framework, the main scientific contribution of the present work is presented in Section 3.2, where the head–shell discontinuity is formulated through displacement compatibility and contour interaction loads. The novelty of the study lies in the integrated analytical–numerical assessment of this junction by correlating the compatibility-based formulation with finite element results and stress linearization according to ASME Section VIII Division 2 [17,18,19,20,21]. This combined approach provides a clear mechanical interpretation of the local stress concentration and of the extent of the discontinuity influence zone.

## 2. Theoretical Framework

### 2.1. Stress Categorization and Decomposition According to ASME VIII Division 2

The structural integrity assessment performed in this study follows the stress categorization framework prescribed by ASME Boiler and Pressure Vessel Code, Section VIII, Division 2, Part 5 (2023 Edition) [5].

The stress state in shell-type structures is described in a local curvilinear coordinate system using meridional (σ_m_), circumferential (σ_t_), and radial (σ_n_) normal stress components. The loading conditions considered are consistent with ASME terminology.

Assuming linear elastic material behavior, the total stress field may be expressed through superposition of individual load contributions (Equation (1)):

(1) $$\sigma =\sigma_{p}+\sigma_{SUS}+\sigma_{EXP}+\sigma_{OCC}$$

This decomposition enables proper classification of stress for strength and fatigue verification.

The subscripts “p”, “SUS”, “EXP”, “OPE” and “OCC” define the different types of stresses that lead to stresses in structural components. These include pressurization, either internal or external, permanent or gravitational loads, together with possible initial failure of the foundations, restricted thermal expansion, stresses specific to the normal regime of service and technological operation, and occasional loads, such as those generated by external factors such as wind or earthquakes.

The characteristic stresses are defined in relation to the local coordinate systems specific to each component and are expressed in three main directions: meridional (m), circumferential or annular (t) and normal (n).

Depending on their nature, these stresses can be:

- Mechanical, caused by external stresses such as pressure, dead weight and occasional loads (“p”, “SUS”, “OCC”);
- Thermo-mechanical, resulting from the constraint of free expansion, which leads to additional stress in the material (“EXP”, “OPE”).
- Thermal, specific to thick-walled structures, where temperature variations create a thermal gradient along the wall thickness (“EXP”).

According to ASME VIII Division 2, Part 5 [5], stresses are categorized into three principal groups: Primary stresses (P); Secondary stresses (Q); Peak stresses (F).

Primary stresses are required to satisfy global force and moment equilibrium and are not self-limiting. Exceeding the yield strength under primary loading may lead to gross plastic deformation or collapse.

Secondary stresses arise from displacement constraints and structural compatibility requirements, such as those induced by geometric discontinuities or restrained thermal expansion. These stresses are self-limiting but may contribute to ratcheting effects under cyclic loading.

Peak stresses represent localized stress intensifications occurring near weld toes, geometric transitions, or discontinuities. Although they do not influence plastic collapse, they are serious in fatigue crack initiation assessment.

For design verification, stresses through the wall thickness are decomposed into membrane and bending components using stress linearization procedures in accordance with ASME VIII Division 2 Part 5. The characteristic extent of local primary stress regions is conventionally estimated as $\sqrt{R\cdot t}$, where R is the local radius of curvature and t is the wall thickness.

### 2.2. Allowable Stress and Strength Verification Criteria

In accordance with ASME Section VIII Division 2, the structural verification aims to prevent plastic collapse, excessive yielding, ratcheting, and fatigue failure under the prescribed loading conditions.

For the present investigation, the design temperature remains within the moderate operating range; therefore, creep-related limit states were not governing and were not explicitly considered in the allowable stress determination.

The allowable design stress $S_{a}$ was determined according to ASME VIII Division 2 provisions based on yield ($S_{y}$) and tensile strength ($S_{u}$) limits at the design temperature:

- Yield-controlled criterion:

(2) $$S_{a}=\frac{S_{y}}{1.5}$$

- Tensile-controlled criterion:

(3) $$S_{a}=\frac{S_{u}}{2.4}$$

The governing allowable stress corresponds to the minimum of the above values. The equivalent stress used for strength verification was evaluated using the Coulomb–Tresca criterion and compared against the hierarchical acceptance limits prescribed by ASME VIII Division 2:

(4) $$P_{m}\leq S_{a}$$

(5) $$P_{m}+P_{b}\leq 1.5S_{a}$$

(6) $$P_{m}+P_{b}+Q\leq 3S_{a}$$

### 2.3. Stress Linearization and Structural Discontinuities

Shell-type pressure vessels frequently contain geometric discontinuities such as thickness transitions, head–shell junctions, and welded connections. These regions introduce local stiffness variations that disturb the nominal membrane stress field and generate additional bending stresses.

According to ASME VIII Division 2, stress evaluation in such regions requires classification along defined Stress Classification Lines (SCLs). Stress linearization separates the total through-thickness stress distribution into:

- Membrane component (uniform across thickness),
- Bending component (linearly varying),
- Peak component (localized nonlinear excess).

The linearized equivalent stress is evaluated using equation:

(7) $$\sigma_{eq}=\sigma_{memb}+\sigma_{bend}$$

while peak stresses are treated separately for fatigue considerations.

In the vicinity of geometric discontinuities, local primary stresses may develop over a characteristic dimension commonly approximated by $\sqrt{R\cdot t}$ where R is the local radius of curvature and t is the wall thickness. Beyond this region, stresses tend to recover toward the nominal membrane state, consistent with Saint-Venant’s principle.

The present study aims to provide a comprehensive structural assessment of the head–shell junction in pressurized railway tank wagons operating under internal pressure, by integrating analytical shell theory with finite element modelling in accordance with ASME Section VIII Division 2 provisions. Attention is devoted to the characterization of stress concentration phenomena generated by geometric discontinuity and displacement compatibility at the ellipsoidal head–cylindrical shell transition. The study seeks to quantify the magnitude and spatial extent of local membrane–bending interaction effects, to evaluate the resulting equivalent stresses using the Coulomb–Tresca criterion, and to verify compliance with the hierarchical stress limits prescribed by the ASME code. Through this combined analytical–numerical framework, the work aims to establish a reliable and code-consistent methodology for the evaluation of structural integrity in discontinuity regions of axisymmetric pressure vessels used in railway transport applications [10].

## 3. Materials and Methods

### 3.1. Structural Configuration and Material Properties

The structural configuration investigated in the present study consists of a rigidly welded junction between a semi-ellipsoidal head (F) and a cylindrical shell (M), representative of pressurized railway tank vessels operating under internal pressure loading conditions. The analyzed assembly forms a thin-walled shell of revolution characterized by a geometric discontinuity at the head–shell transition contour. This discontinuity results from the difference in curvature and bending stiffness between the ellipsoidal head and the cylindrical shell.

Under uniform internal pressurization, each structural component tends to deform according to its own membrane stress state. However, because the head and the shell are rigidly connected along the common equatorial contour $\Gamma_{0}$, displacement compatibility must be satisfied. Consequently, the junction region exhibits stress redistribution and local bending effects superimposed on the nominal membrane stresses.

**Figure 1:**
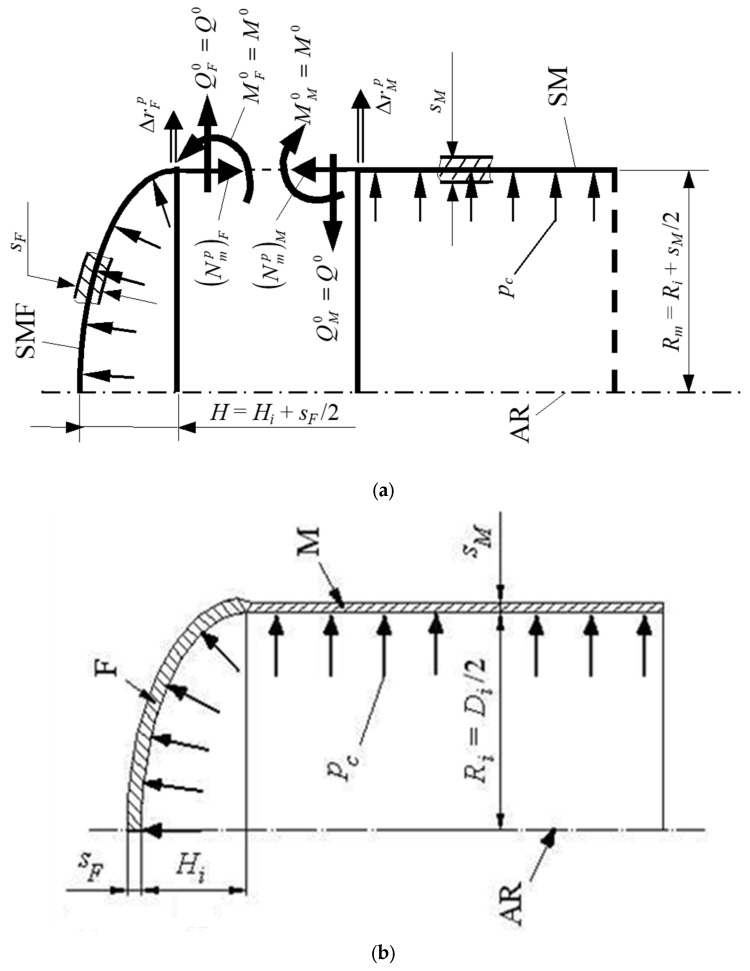
Geometry and loading configuration of the semi-ellipsoidal head–cylindrical shell (F–M) junction under uniform internal pressure. (**a**) loading configuration; (**b**) schematic representation of the junction.

The adopted geometric configuration and material properties correspond to a 60 m^3^ rail tank wagon designed for internal pressure service [22]. The principal geometric and mechanical parameters used in the analytical and numerical investigation are summarized in Table 1, while the schematic representation of the junction and loading configuration is illustrated in Figure 1.

The analysis was conducted under a uniform internal pressure loading of 0.45 MPa, assuming linear-elastic material behavior and small deformation conditions.

The pressure vessel investigated in the present study is manufactured from R44.5a steel, a carbon–manganese structural steel traditionally used in the fabrication of railway tank wagons in Romania. The material is specified in the Romanian standard STAS 2883/2 [23], which defines steels intended for welded pressure-containing structures subjected to moderate internal pressure.

For the purpose of the analytical and numerical investigation, the material was modelled as a homogeneous, isotropic, linear-elastic steel, characterized by the mechanical properties listed in Table 1: Young’s modulus E=190,000 MPa, Poisson’s ratio ν=0.30, yield strength $S_{y}=308$ MPa, and ultimate tensile strength $S_{u}=577$ MPa. These values correspond to the typical strength range of carbon–manganese steels historically used in welded pressure vessels and railway tank structures.

From a historical standards perspective, steel R44.5a is closely related to the German pressure-vessel steel ASt 45, specified in DIN 17135 [24] and referenced in SEW 089-70 [25] and SEW 680-70 [26], which were widely used for welded boiler and pressure-vessel components. These steels belong to the same class of weldable carbon–manganese steels designed for moderate pressure applications.

### 3.2. Analytical Formulation of the Head–Shell Junction

The analytical formulation of the head–shell discontinuity is developed within the framework of the classical moment theory for thin shells of revolution, applicable to axisymmetric thin-walled structures subjected to internal pressure loading.

To justify the application of classical shell theory, the thin-shell condition was verified. For the cylindrical shell, the thickness-to-radius ratio is:

(8) $$\frac{s_{M}}{R_{m}}=\frac{8}{1404}\approx 0.0057$$

and for the ellipsoidal head:

(9) $$\frac{s_{F}}{R_{m}}=\frac{10}{1404}\approx 0.0071$$

Both ratios are significantly lower than the conventional thin-shell limit t/R<0.1, confirming that membrane and bending behavior may be accurately described using classical moment theory for shells of revolution. Under these geometric conditions, transverse shear deformation effects are negligible and the stress distribution across the wall thickness may be assumed linear in bending and uniform in the membrane regime.

The analytical evaluation of the head–shell junction is performed using the classical theory of shells of revolution with moments, applicable to axisymmetric thin-walled structures subjected to internal pressure. In the free membrane regime, each component develops meridional and circumferential membrane forces and corresponding radial displacements at the junction contour. These free radial displacements are denoted $\Delta r_{F}^{p}$ for the ellipsoidal head and $\Delta r_{M}^{p}$ for the cylindrical shell. Owing to the difference in geometry and stiffness between the two components, these quantities are generally not equal.

In the free membrane regime, each component develops its own radial displacement at the junction contour. Due to the difference in geometry and stiffness of the two connected components, in this case the ellipsoidal head and the cylindrical shell, the free radial displacements at the junction are generally different; therefore, compatibility must be enforced through Equations (10) and (11). Because the bottom (F) and the mantle (M) are rigidly connected at the equatorial parallel $\Gamma_{0}$, the actual radial displacements and rotations must satisfy the following compatibility conditions:

(10) $$\Delta r_{F}=\Delta r_{M}$$

(11) $$\theta_{F}=\theta_{M}$$

To enforce these conditions, uniformly distributed contour loads are introduced along the junction contour: a meridional bending moment per unit length $M_{0}$ [(N·mm)/mm] and a transverse shear force per unit length $Q_{0}$ [N/mm]. Here, Δr denotes the radial displacement and θ the rotation at the junction contour, while the subscripts F and M refer to the ellipsoidal head and the cylindrical shell, respectively. The coefficients δ and η represent the displacement and rotation influence coefficients associated with unit contour loads.

These loads superpose the membrane solution and modify the displacement and rotation fields according to the principle of superposition.

The total radial displacements and rotations at the contour are therefore expressed as:

(12) $$\Delta r_{F}=\Delta r_{F}^{p}+\delta_{F}^{M_{0}}M_{0}+\delta_{F}^{Q_{0}}Q_{0}$$

(13) $$\Delta r_{M}=\Delta r_{M}^{p}+\delta_{M}^{M_{0}}M_{0}+\delta_{M}^{Q_{0}}Q_{0}$$

(14) $$\theta_{F}=\theta_{F}^{p}+\eta_{F}^{M_{0}}M_{0}+\eta_{F}^{Q_{0}}Q_{0}$$

(15) $$\theta_{M}=\theta_{M}^{p}+\eta_{M}^{M_{0}}M_{0}+\eta_{M}^{Q_{0}}Q_{0}$$

Imposing displacement and rotational compatibility yields the system:

(16) $$\Delta r_{F}-\Delta r_{M}=0$$

(17) $$\theta_{F}-\theta_{M}=0$$

which leads to the linear algebraic system in the unknown contour loads $M_{0}$ and $Q_{0}$:

(18) $$\left(\delta_{M}^{M_{0}}-\delta_{F}^{M_{0}})M_{0}+(\delta_{M}^{Q_{0}}-\delta_{F}^{Q_{0}})Q_{0}=-(\Delta r_{M}^{p}-\Delta r_{F}^{p}\right)$$

(19) $$\left(\eta_{M}^{M_{0}}-\eta_{F}^{M_{0}})M_{0}+(\eta_{M}^{Q_{0}}-\eta_{F}^{Q_{0}})Q_{0}=-(\theta_{M}^{p}-\theta_{F}^{p}\right)$$

The coefficients *⸹* and *η* represent displacement and rotation responses induced by unit contour loads and are determined according to the classical moment theory of shells of revolution.

The evaluation of these influence coefficients follows the standard analytical formulation for axisymmetric shell discontinuities, as presented in classical shell theory references [27,28,29].

These relations represent the explicit formulation of displacement and rotation compatibility at the head–shell junction and form the basis for determining the contour interaction loads that govern the local membrane–bending coupling in the discontinuity region.

The values of $Q_{0}$ and $M_{0}$ are obtained by solving the linear algebraic system defined by Equations (18) and (19), which follows from the compatibility conditions imposed at the rigidly connected head–shell junction. For the adopted geometry, material properties, and internal pressure of 0.45 MPa, the solution gives $Q_{0}=795$ N/mm and $M_{0}=13,350$ (N·mm)/mm. These quantities represent the transverse shear force and bending moment per unit length required to compensate for the mismatch of free deformation between the ellipsoidal head and the cylindrical shell.

These interaction forces generate additional stress resultants in both structural components. The meridional and circumferential normal stresses are expressed by combining membrane forces and bending moments as:

(20) $${\left(\sigma_{t}^{M^{0}+Q^{0}}\right)}_{F}^{+,-}\left(\phi \right)=\frac{{\left(N_{t}^{M^{0}+Q^{0}}\right)}_{F}\left(\phi \right)}{s_{F}}\pm \frac{6\cdot {\left(M_{t}^{M^{0}+Q^{0}}\right)}_{F}\left(\phi \right)}{s_{F}^{2}}$$

(21) $${\left(\sigma_{t}^{M^{0}+Q^{0}}\right)}_{M}^{+,-}\left(x\right)=\frac{{\left(N_{t}^{M^{0}+Q^{0}}\right)}_{M}\left(x\right)}{s_{M}}\pm \frac{6\cdot {\left(M_{t}^{M^{0}+Q^{0}}\right)}_{M}\left(x\right)}{s_{M}^{2}}$$

The total stress field is obtained by superposition of membrane stresses induced by internal pressure and additional stresses generated by $M_{0}$ and $Q_{0}$:

(22) $${\left(\sigma_{t}\right)}_{F}^{+,-}\left(\phi \right)={\left(\sigma_{t}^{p}\right)}_{F}\left(\phi \right)+{\left(\sigma_{t}^{M^{0}+Q^{0}}\right)}_{F}^{+,-}\left(\phi \right)$$

(23) $${\left(\sigma_{t}\right)}_{M}^{+,-}\left(x\right)={\left(\sigma_{t}^{p}\right)}_{M}\left(x\right)+{\left(\sigma_{t}^{M^{0}+Q^{0}}\right)}_{M}^{+,-}\left(x\right)$$

The equivalent stress at the inner and outer surfaces is evaluated using the maximum shear stress criterion:

(24) $${\left(\sigma_{ech}\right)}_{M}^{+,-}\left(x\right)=\max\left\{\left|{\left(\sigma_{m}\right)}_{M}^{+,-}\left(x\right)\right|,\left|{\left(\sigma_{t}\right)}_{M}^{+,-}\left(x\right)\right|,\left|{\left(\sigma_{m}\right)}_{M}^{+,-}\left(x\right)-{\left(\sigma_{t}\right)}_{M}^{+,-}\left(x\right)\right|\right\}$$

${\left(\sigma_{ech}\right)}_{M}^{+,-}\left(x\right)$ is the resultant equivalent normal mechanical stress, at the level of the boundary surfaces (inner, SI⟶”+” and outer SE⟶”-“) of the wall M, corresponding to the current distance x associated with the current parallel Γ_x_ belonging to SM, expressed in N/mm^2^.

These analytical expressions define the stress state in the discontinuity region of the head–shell junction and constitute the theoretical basis for subsequent numerical validation using finite element analysis.

### 3.3. Finite Element Modeling of the Railway Tank Wagon Discontinuity Zone

**Figure 2:**
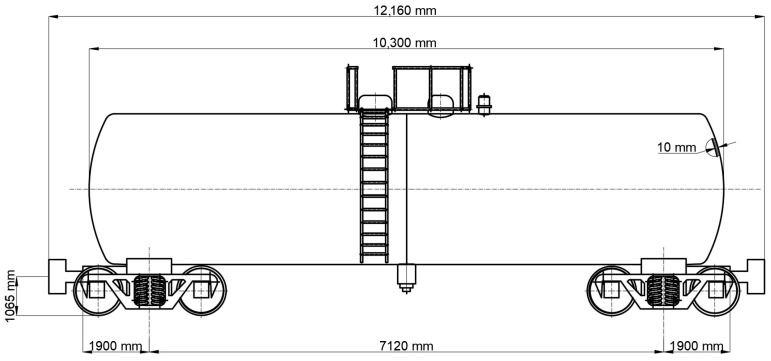
Schematic representation of the tank wagon.

The numerical investigation was carried out on a 60 m^3^ cylindrical railway tank wagon equipped with semi-ellipsoidal end caps, representative of pressurized vessels used for the transportation of liquefied gases and chemical products. The global configuration of the tank wagon is illustrated in Figure 2. Structurally, the vessel consists of a welded cylindrical shell closed by ellipsoidal bottoms, forming a shell of revolution characterized by geometric discontinuity at the head–shell junction.

The finite element model was developed using ANSYS 2025 R2 software to complement the analytical formulation and to provide an independent evaluation of the stress state in the discontinuity region. The numerical model reproduces the geometric configuration, material properties, and loading conditions adopted in the analytical approach.

The principal geometric parameters correspond to an internal diameter of 2800 mm, cylindrical shell thickness of 8 mm, ellipsoidal head thickness of 10 mm, and an internal design pressure of 0.45 MPa. The cylindrical length of the tank is 8750 mm. These dimensions reflect the actual structural configuration of the investigated tank wagon.

The material was modeled as homogeneous, isotropic, and linearly elastic, characterized by a Young’s modulus E=190,000 MPa, Poisson’s ratio ν=0.30, and yield strength of 308 MPa. The numerical analysis was performed within the small-deformation regime, neglecting geometric nonlinearity, plasticity effects, and time-dependent phenomena, in accordance with the assumptions adopted in the analytical shell theory formulation.

Given the axial symmetry of both geometry and loading, an axisymmetric modeling strategy was employed to ensure computational efficiency while preserving the physical consistency of the stress field in the vicinity of the head–shell junction. At the same time, a three-dimensional representation of the tank was implemented to capture the global structural response and to verify the validity of the simplified axisymmetric approach. The connection between the cylindrical shell and the ellipsoidal head was modeled as perfectly bonded, ensuring displacement continuity along the common junction contour.

Uniform internal pressure p=0.45 MPa was applied to the inner surfaces of both the cylindrical shell and the ellipsoidal heads. Boundary conditions were defined to eliminate rigid-body motion while avoiding artificial stiffness in the discontinuity region. The model length in the cylindrical direction was selected such that the stress field decays toward the nominal membrane state away from the junction, allowing the identification of the effective influence zone of the geometric discontinuity.

The numerical analysis was carried out in two distinct versions, each with specific purposes and methods, which allowed a detailed assessment of the structural behavior of the tank. These approaches were adopted to ensure a comprehensive evaluation of the stress state using both three-dimensional (3D) and planar (2D) modeling techniques.

**Figure 3:**
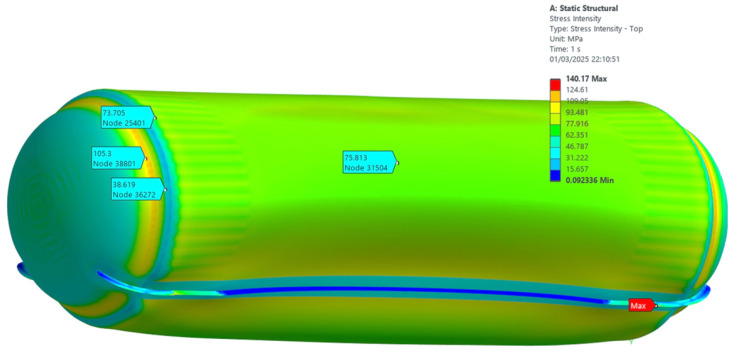
Equivalent stress distribution at the head–shell junction obtained from the 3D finite element model, evaluated according to the Coulomb–Tresca criterion.

1. The 3D modeling in shell theory (Figure 3): This approach was used to obtain a global view of the behavior of the structure under external stresses. The 3D model was designed to analyze the distribution of stresses and strains throughout the entire structure, considering the influences of specific constraint conditions, such as those in the studied zones.

It allowed a detailed representation of the structure, including the interactions between the different tank components, as well as the effects of the applied loads on the whole geometry. Thus, the analysis has contributed to the understanding of how the areas of drag influence the structural behavior, having a significant impact on the stress distribution throughout the structure.

**Figure 4:**
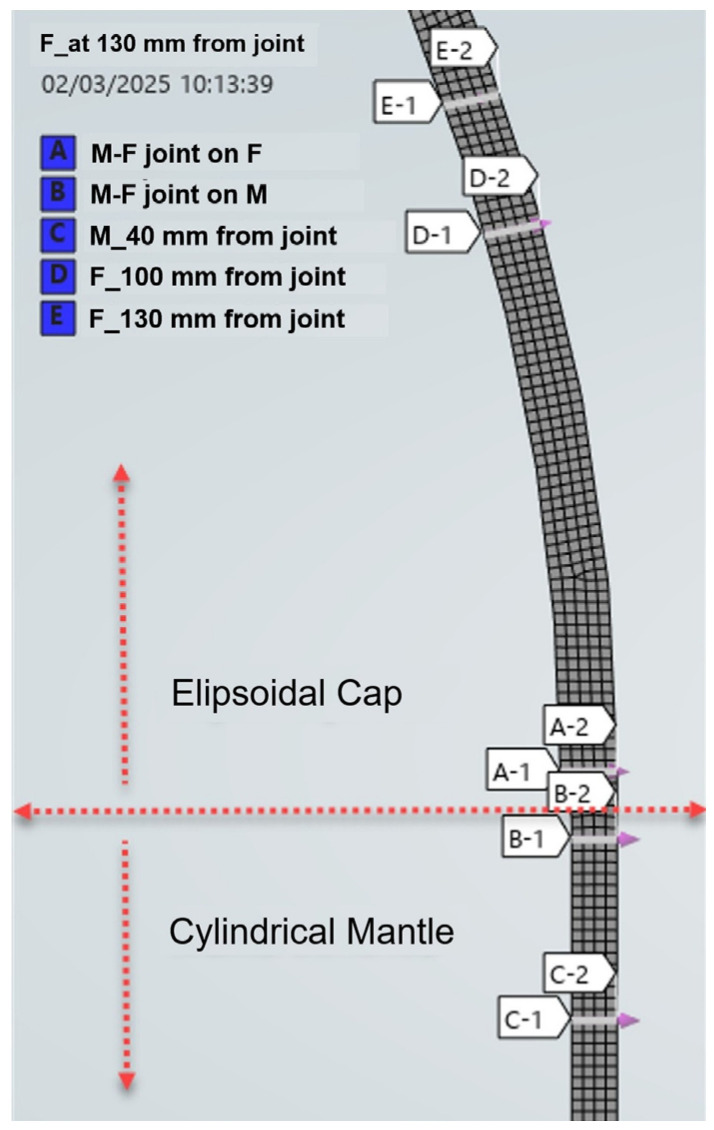
Stress Classification Lines (SCLs) used for stress linearization and code-based verification.

2. Planar analysis model (2D-Figure 4): In this variant of analysis, a more simplified approach was used, which allowed a detailed examination of the local behavior of the structure.

The planar analysis model was designed to quantify and interpret stress states in a specific section of the tank. This approach was useful to evaluate the effects of loading on specific areas such as joints or risky areas. The 2D model was also applied in accordance with the precepts of the relevant design codes, namely ASME VIII Division 2 and AS1210 [6], providing a solid basis for validating the results and ensuring compliance with international standards.

A significant advantage of the 2D analysis was the possibility to compare the numerical results with standard analytical solutions. This comparison allowed a rigorous validation of the numerical model and provided insight into its accuracy and reliability under given loading conditions. The 2D analysis was also essential for a better understanding of local stress concentrations and structural behaviour in the critical areas of the vessel.

A locally refined mesh was introduced in the vicinity of the head–shell junction to accurately capture the steep stress gradients associated with bending and transverse shear effects induced by displacement compatibility constraints. Outside the transition region, a progressively coarser mesh was employed to optimize computational efficiency without compromising numerical accuracy.

A mesh convergence study was performed to ensure that the numerical results are independent of the mesh density. Since the highest stress gradients occur near the geometric discontinuity at the head–shell junction, local mesh refinement was applied in this region, while a progressively coarser mesh was used away from the transition zone to maintain computational efficiency. Three progressively refined meshes were analyzed, corresponding to characteristic element sizes of approximately 12 mm, 8 mm, and 5 mm in the junction region. Convergence was evaluated by monitoring both the maximum equivalent stress and the linearized stresses along the critical Stress Classification Lines. The difference between the intermediate and the finest mesh remained below 2% for the maximum equivalent stress, while the membrane and bending stress components obtained through stress linearization showed negligible additional variation.

The refined mesh was therefore adopted for all subsequent stress evaluation and stress linearization procedures.

The axisymmetric finite element model was discretized using four-node quadrilateral structural elements (ANSYS PLANE182) with quadratic displacement interpolation. Numerical integration was performed using the standard Gauss integration scheme implemented in the ANSYS element formulation. The three-dimensional model was discretized using four-node shell elements (ANSYS SHELL181), which employ first-order shear deformation theory and allow accurate representation of membrane and bending behavior in thin-walled structures. These elements use reduced integration with hourglass control to ensure numerical stability while maintaining computational efficiency.

The finite element analysis provides the full stress tensor distribution throughout the structure. For direct comparison with the analytical predictions and for strength verification in accordance with the applicable design codes, stress extraction and linearization procedures were carried out along predefined Stress Classification Lines (SCLs) located in the head–shell discontinuity region and in the adjacent shell areas, as illustrated in Figure 4.

**Figure 5:**
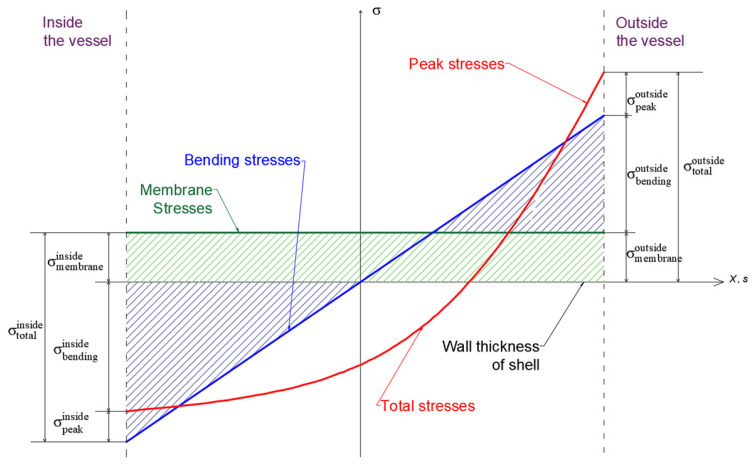
Schematic representation of stress linearization into membrane, bending, and peak components along the wall thickness.

The stress decomposition concept adopted for linearization, including the separation into membrane, bending, and peak components through the wall thickness, is schematically presented in Figure 5. The defined SCLs were subsequently used to evaluate membrane stresses, bending stresses, and their combined contributions, forming the basis for the code-compliant structural integrity assessment.

For code-based verification, stresses were extracted along predefined Stress Classification Lines (SCLs) passing through the wall thickness at critical locations near the head–shell junction. The stress tensor obtained from the finite element solution was sampled at multiple points along each SCL, and the through-thickness stress distribution was subsequently processed using the ASME Section VIII Division 2 stress linearization procedure.

Linearization was applied to the stress tensor components along the local coordinate directions, allowing the membrane and bending stress components to be determined prior to evaluation of equivalent stress measures. This procedure ensures consistency with the tensor-based stress linearization requirements prescribed by ASME Division 2 for design-by-analysis verification.

The finite element model therefore constitutes an independent validation framework for the analytical solution and enables a detailed quantification of local stress concentration phenomena in the head–shell transition region of railway tank wagons subjected to internal pressure loading.

## 4. Results and Discussion

### 4.1. Analytical Results

The analytical stress field in the head–shell discontinuity zone was obtained by solving the compatibility equations derived in Section 2.2 using a dedicated MATLAB R2023b-based computational routine. The system of linear equations associated with the contour interaction loads $M_{0}$ and $Q_{0}$ was solved numerically, and the resulting stress components were evaluated along the meridional direction of both the cylindrical shell (M) and the ellipsoidal head (F).

**Figure 6:**
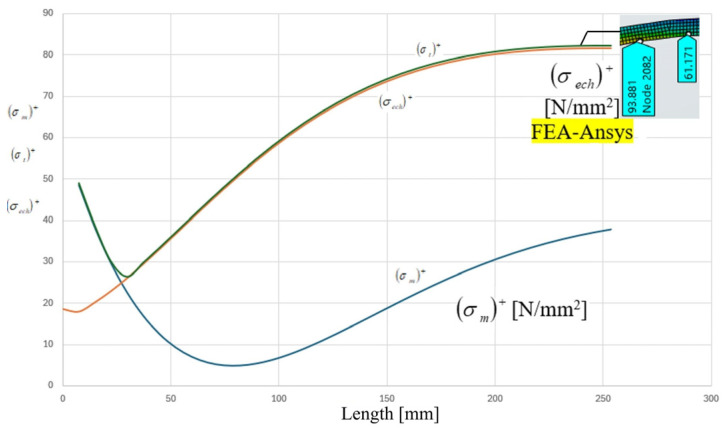
The distributions of the resultant mechanical stresses, recorded along the meridian of the cylindrical component M, starting from the junction area, over 300 mm from the contour-interior of the junction.

**Figure 7:**
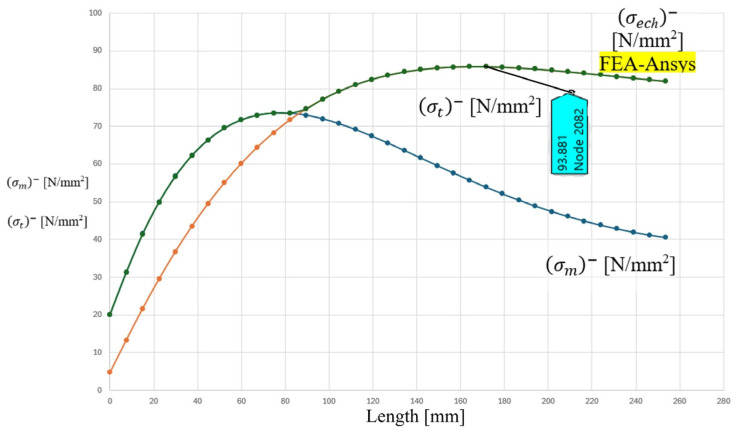
The distributions of the resultant mechanical stresses, recorded along the meridian of the cylindrical component M, starting from the junction area, over 300 mm from the contour-outside of the junction.

The analytical distributions of meridional stress $\sigma_{m}$, circumferential stress $\sigma_{t}$, and equivalent stress $\sigma_{eq}$ along the meridians of the cylindrical shell are presented in Figure 6 and Figure 7. The coordinate origin corresponds to the head–shell junction contour, and the stress evolution is recorded over a distance of 300 mm from the junction.

In the immediate vicinity of the junction (x ≈ 0), a pronounced perturbation of the nominal membrane stress state is observed. For the cylindrical shell, the meridional stress $\sigma_{m}$ exhibits a local minimum close to the contour, followed by a gradual recovery toward the membrane-dominated state. Conversely, the circumferential stress $\sigma_{t}$ increases rapidly from the junction and stabilizes at a nearly constant level at distances exceeding approximately 120–150 mm from the contour.

The equivalent stress $\sigma_{eq}$, evaluated according to the maximum shear stress criterion, reaches its maximum value at or very close to the junction contour. Based on the graphical evaluation of the analytical curves, the peak equivalent stress in the cylindrical component is approximately 80–85 MPa, while the nominal membrane stress away from the discontinuity stabilizes at approximately 70–75 MPa. This indicates a moderate but clearly identifiable stress amplification induced by geometric incompatibility.

**Figure 8:**
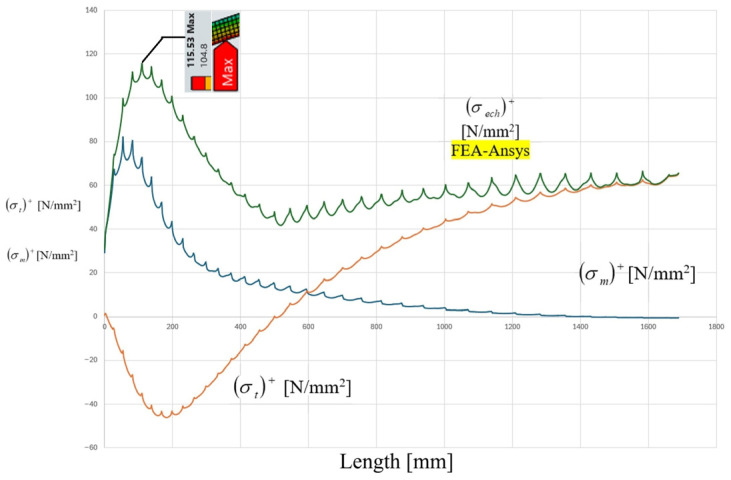
The distributions of the mechanical resultant stresses, recorded along the meridian of the F-component, starting from the junction zone to the INNER-pole of the junction.

**Figure 9:**
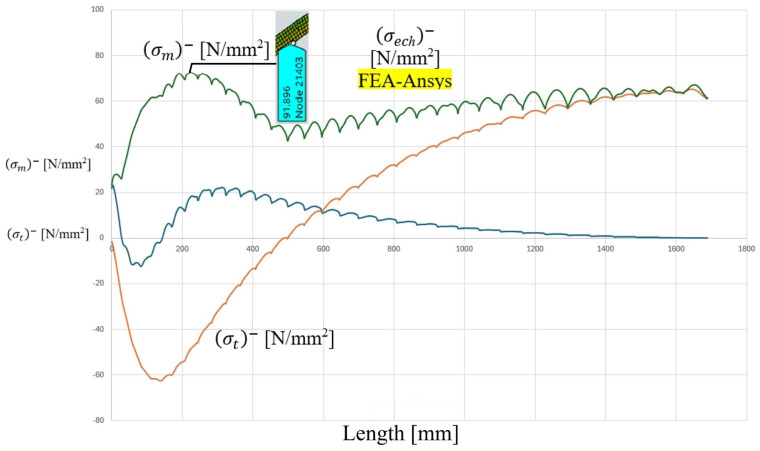
The distributions of the mechanical resultant stresses, recorded along the meridian of component F, starting from the junction area to the EXTERIOR-JOINT pole.

A similar behavior is observed on the ellipsoidal head side, as illustrated in Figure 8 and Figure 9. However, the stress concentration effect is more pronounced in the head component. The analytical solution predicts a sharper stress gradient in the vicinity of the contour, followed by a gradual attenuation toward the interior pole of the head. The maximum equivalent stress in the head region is estimated to be slightly higher than that of the cylindrical shell, approaching values in the range of 90 MPa based on the graphical representation.

The analytical results clearly indicate that the discontinuity-induced bending and shear effects are localized within a finite influence zone. Beyond approximately 120–150 mm from the junction contour, the stress distribution converges toward the nominal membrane solution corresponding to uniform internal pressurization. This observation is consistent with classical shell theory, according to which the effect of local discontinuities decays over a characteristic length proportional to $\sqrt{R\cdot t}$.

From a mechanical standpoint, the stress concentration at the head–shell junction is primarily governed by the incompatibility of radial displacements between the two shell segments. The interaction forces $M_{0}$ and $Q_{0}$ introduced to enforce compatibility generate additional bending moments in the vicinity of the contour, leading to local amplification of meridional and circumferential stresses. The analytical model therefore confirms that the head–shell junction represents a structurally sensitive region, where stress redistribution effects must be carefully evaluated in the context of design verification.

These analytical findings establish the theoretical baseline for subsequent numerical validation and provide insight into the magnitude and spatial extent of stress concentration phenomena in railway tank wagons subjected to internal pressure loading.

### 4.2. Numerical Results (Finite Element Analysis)

The finite element analysis provided a detailed representation of the stress field in the head–shell discontinuity region under internal pressure loading of 0.45 MPa.

**Figure 10:**
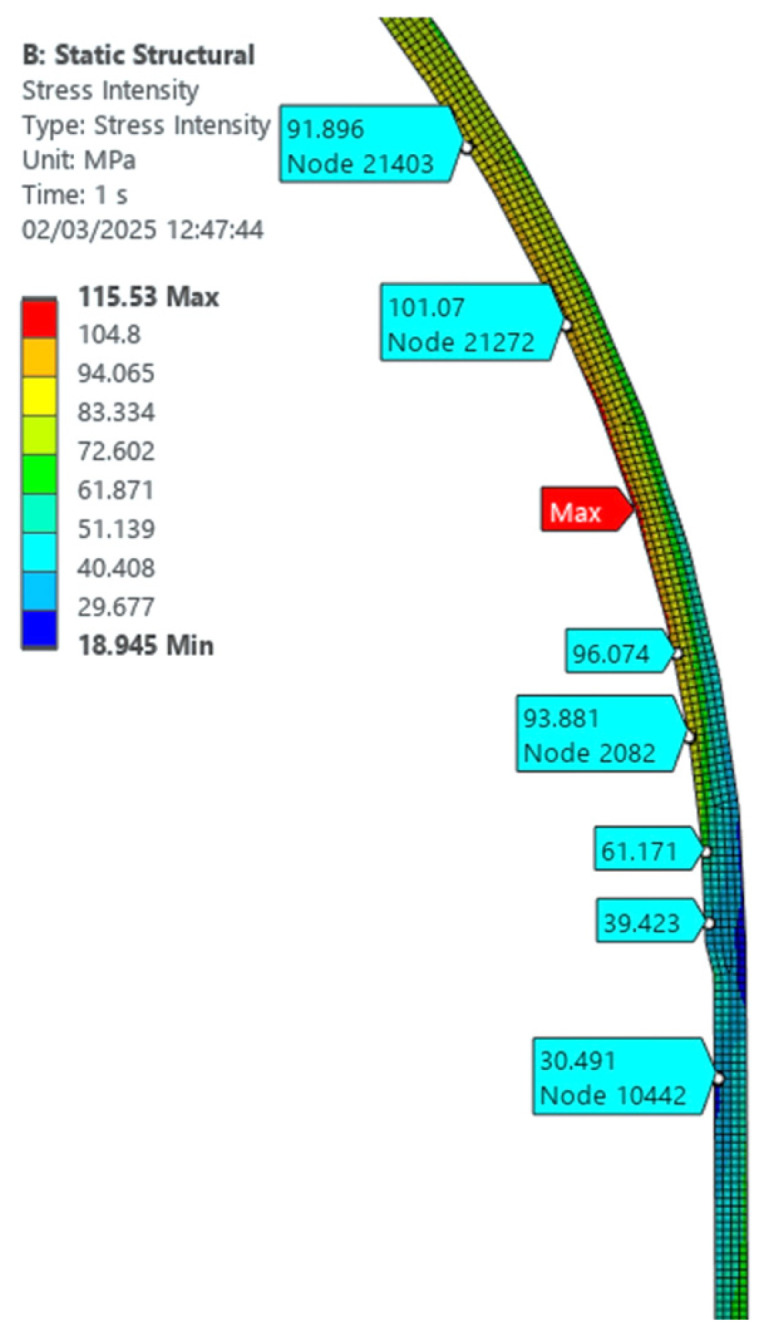
Equivalent stress distribution at the head–shell junction obtained from the axisymmetric 2D finite element model, evaluated according to the Coulomb–Tresca criterion.

It can be seen from Figure 3 and Figure 10 that the maximum stress develops at the junction between the shell and the support system. Outside this region, the maximum stress is observed in the vicinity of the junction contour between the cylindrical component (the mantle) and the ellipsoidal component (the ellipsoidal cap). More precisely, this maximum stress is manifested on the ellipsoidal cap at a distance of about 130 mm, measured on its generators.

The comparative analysis of the two modeling methods shows a good agreement between the obtained results. According to the Coulomb-Tresca criterion, the maximum stress determined by the axial-symmetric solid model approach is about 115 MPa, while the shell finite element modeling indicates a slightly higher value of 117 MPa.

This correlation between the two analysis methods underlines the validity of the approaches used and confirms that the stress distribution in the analyzed structure is correctly estimated.

The stress distribution along the structure indicates that away from the discontinuity region, the stress field gradually approaches the nominal membrane state characteristic of uniform internal pressure. This confirms that the junction behaves as a localized disturbance superimposed on the global membrane stress regime.

The axisymmetric (2D) finite element model provides a refined description of the local stress distribution in the discontinuity zone. The equivalent stress map obtained from the planar model is shown in Figure 10. The maximum equivalent stress predicted by the 2D model is approximately 115 MPa, occurring in the same region identified in the 3D simulation.

The comparison between the two numerical approaches demonstrates very good agreement, with differences below approximately 2 MPa, confirming the adequacy of the axisymmetric formulation for evaluating stress concentration phenomena in geometrically axisymmetric structures subjected to internal pressure.

Both numerical models indicate that the highest stress concentration occurs on the ellipsoidal head rather than on the cylindrical shell, highlighting the influence of curvature transition and compatibility constraints at the head–shell junction. The stress gradient is particularly steep within the first 100–150 mm from the contour, after which the stress field stabilizes toward the nominal membrane level.

These results confirm that the geometric discontinuity induces localized bending effects superimposed on membrane stresses, consistent with the predictions obtained from the analytical formulation based on classical shell theory.

In order to assess structural integrity in accordance with ASME Section VIII Division 2, stress linearization was performed along the predefined Stress Classification Lines (SCLs) illustrated in Figure 5.

In accordance with ASME Section VIII Division 2, stress linearization was performed on the full stress tensor obtained from the finite element analysis, extracted along each Stress Classification Line through the wall thickness. The linearization procedure was applied component-wise to the stress tensor prior to the evaluation of principal stresses or equivalent stress measures. The through-thickness stress distribution was decomposed into membrane and bending components according to the standard ASME linearization procedure, yielding the primary membrane stress $P_{m}$ and the primary bending stress $P_{b}$. Secondary stresses Q were identified from the deviation of the total stress field from the linearized membrane–bending distribution.

The structural verification was then performed using the hierarchical limits defined by ASME Section VIII Division 2, namely $P_{m}$, $P_{m}+P_{b}$, and $P_{m}+P_{b}+Q$. It should be noted that the Coulomb–Tresca equivalent stress was used only to visualize and compare global stress intensity distributions within the structure. The code compliance assessment itself was based on the linearized membrane and bending stress components extracted along the Stress Classification Lines, in accordance with the tensor-based linearization requirements of ASME Division 2.

The evaluated sections include: SCL1 (A1–A2) at the head–shell junction on the ellipsoidal cap side; SCL2 (B1–B2) at the head–shell junction on the cylindrical shell side; SCL3 (E1–E2) located on the ellipsoidal head at approximately 130 mm from the junction contour; and SCL4 (C1–C2) positioned on the cylindrical shell at approximately 40 mm from the junction.

For each SCL, membrane stresses (P_m_), primary bending stresses (P_b_), and their linearized combinations were extracted from the finite element results and compared with the allowable stress limits prescribed by ASME VIII Division 2. The verification results are summarized in Table 2.

As shown in Table 2, the highest combined local membrane and bending stress was obtained at SCL3 (E1–E2), reaching 109.5 MPa. This value remains significantly below the allowable limit of 308 MPa for primary stress categories and 616 MPa for combined stress categories in accordance with ASME VIII Division 2.

It should be noted that the quantities reported in Table 2 correspond to stress intensities obtained from the stress tensors resulting from the ASME stress linearization procedure. In accordance with ASME BPVC Section VIII Division 2, the stress linearization is performed on the full stress tensor extracted along the Stress Classification Line (SCL).

The membrane stress Pm is obtained as the through-thickness average of the stress tensor components along the SCL. The primary bending stress Pb is determined from the linear variation of the stress tensor relative to the membrane component. The combined quantity Pm + Pb corresponds to the stress tensor obtained by superposition of the membrane and bending tensors. The scalar stress intensity reported in the table is then evaluated from this combined tensor.

Consequently, the reported values of Pm, Pb, and Pm + Pb do not represent simple algebraic sums of scalar quantities. Instead, they correspond to stress intensities derived from the respective linearized stress tensors, which explains why the value associated with Pm + Pb is not necessarily equal to the arithmetic sum of the individually listed membrane and bending stress intensities.

In the stress linearization procedure, P_m_ denotes the membrane stress component, *P_b_* the primary bending stress component, and Q the additional peak/secondary contribution considered in the combined stress evaluation. The symbols *S_y_* and *S_u_* denote the yield strength and ultimate tensile strength of the material, respectively.

All evaluated stress combinations satisfy the code acceptance criteria, confirming that the head–shell junction meets the structural integrity requirements under the considered internal pressure loading of 0.45 MPa.

### 4.3. Comparative Assessment (Analytical vs. FEA)

The analytical formulation based on classical shell theory and moment compatibility provides a closed-form estimation of stress amplification in the head–shell discontinuity region. The finite element analysis, performed using both axisymmetric (2D) and three-dimensional (3D) models, offers a numerical representation of the complete stress tensor distribution under identical loading and material assumptions.

From the analytical solution, the maximum equivalent stress in the discontinuity zone was estimated to be in the range of approximately 85–90 MPa, depending on the considered location and stress component combination. The analytical approach captures the membrane–bending interaction induced by geometric incompatibility and predicts a localized stress amplification near the junction contour, with gradual attenuation away from the transition region.

In contrast, the finite element simulations yielded maximum equivalent stresses of approximately 115 MPa (2D axisymmetric model, Figure 10) and 117 MPa (3D model, Figure 3). The relative deviation between the 2D and 3D numerical solutions is approximately 1.7%, confirming the consistency of the numerical formulation and the adequacy of the axisymmetric representation for this geometrically symmetric configuration.

When comparing the analytical and numerical results, the finite element method predicts higher peak stresses than the analytical solution. Considering the conservative 3D value (117 MPa) and an analytical estimate of 90 MPa, the relative difference may be expressed as:

(25) $$\Delta (\%)=\frac{117-90}{117}\times 100\approx 23\%$$

This difference can be attributed to several factors. First, the analytical solution is based on classical linear shell theory with idealized boundary assumptions and simplified stress decomposition, which inherently smooths local stress concentrations. Second, the finite element model captures localized three-dimensional stress effects, including stress redistribution near geometric discontinuities, which are not fully represented in the closed-form formulation. Third, numerical stress extraction inherently reflects local mesh resolution and peak stress sensitivity, especially in regions with steep gradients.

A more detailed examination of the stress components indicates that the main source of discrepancy between the analytical and numerical predictions is associated with the bending contribution in the vicinity of the head–shell junction.

The analytical formulation, based on classical Love–Kirchhoff thin-shell theory, assumes a linear distribution of stresses through the thickness and neglects transverse shear deformation effects. Under these assumptions, bending stresses are evaluated from curvature changes predicted by the shell theory solution. In contrast, the finite element model captures the full three-dimensional stress redistribution occurring in the junction region, including localized bending amplification induced by the curvature transition between the cylindrical shell and the ellipsoidal head.

The comparison of stress components shows that the membrane stresses predicted by the analytical formulation remain close to the corresponding finite element values away from the discontinuity zone, while the bending stresses predicted by the numerical model are higher in the immediate vicinity of the junction. This difference reflects the influence of local three-dimensional stress redistribution effects that are not fully represented in the classical thin-shell formulation.

Furthermore, the finite element model captures localized stress intensification associated with curvature gradients and geometric discontinuity effects, which tend to increase the peak stress values extracted from the numerical solution. Since the analytical formulation represents an idealized shell response with smooth curvature transitions, it naturally produces lower peak stresses than the numerical solution.

Therefore, the observed difference between analytical and numerical peak equivalent stresses should primarily be interpreted as the result of localized bending amplification and three-dimensional stress redistribution captured by the finite element model, rather than as a fundamental inconsistency of the analytical formulation.

Despite this quantitative difference in peak values, both approaches consistently identify the head–shell junction as the critical stress region. The ellipsoidal head side is the location of maximum equivalent stress; a localized disturbance zone also extends approximately 120–150 mm from the junction contour; and a progressive decay of stresses moves toward the nominal membrane state away from the discontinuity.

Therefore, the analytical solution provides a reliable qualitative prediction of stress concentration mechanisms and structural behavior, while the finite element analysis offers a more detailed and conservative quantification of peak stress magnitudes.

The overall agreement between the two methods validates the theoretical framework adopted in this study and confirms that classical shell theory remains a valuable tool for preliminary design and interpretation of stress concentration phenomena in axisymmetric pressure vessels. At the same time, finite element modeling is essential for the accurate quantification of local stress maxima in geometrically discontinuous regions.

Similar differences between analytical thin-shell solutions and finite element predictions near shell discontinuities have also been reported in the literature for head–shell and nozzle junctions in pressure vessels.

## 5. Conclusions

The stress state in the head–shell discontinuity region of a pressurized railway tank wagon was investigated through a combined analytical–numerical approach integrating classical shell theory with finite element analysis interpreted according to the ASME Section VIII Division 2 stress linearization methodology.

The analytical solution, based on classical moment theory for shells of revolution, yielded interaction contour loads of Q_0_ = 795 N/mm and M_0_ = 13,350 (N·mm)/mm, confirming the presence of bending effects induced by displacement compatibility at the junction. These interaction forces generate additional stresses superimposed on the nominal membrane stresses in the transition region.

The analytical formulation explicitly introduces displacement and rotation compatibility conditions at the head–shell junction, allowing the contour interaction loads governing the discontinuity response to be determined in a transparent mechanical form. This formulation provides a direct link between classical shell theory predictions and the membrane–bending stress components used in modern code-based structural verification.

Finite element analysis showed that the maximum equivalent stress occurs on the ellipsoidal head at approximately 130 mm from the junction, reaching 115 MPa (2D model) and 117 MPa (3D model). The difference between the two approaches is below 2%, demonstrating that the axisymmetric formulation provides accurate stress prediction for geometrically symmetric configurations.

Stress linearization according to ASME VIII Division 2 indicated that the maximum combined primary membrane and bending stress (109.5 MPa, SCL3) remains well below the allowable limit of 308 MPa, while the extended limit for combined stress categories (616 MPa) is not approached in any evaluated section.

These results confirm that the investigated head–shell configuration satisfies ASME structural integrity requirements under the specified design pressure and that the combined analytical–numerical methodology provides a reliable framework for the assessment of stress concentration phenomena in railway tank wagons.

## Figures and Tables

**Table 1:** Geometric and material parameters of the investigated F–M junction.

Parameter	Symbol	Value
Internal diameter of cylindrical shell	D_i_	2800 mm
Mean radius of cylindrical shell	R_m_	1404 mm
Wall thickness of shell	S_M_	8 mm
Wall thickness of head	S_F_	10 mm
Internal height of head	H_i_	700 mm
Mean head height	H	702 mm
Ellipticity coefficient	k_e_ = a/b	2
Calculation length	L	2100 mm
Young’s modulus	E	190,000 MPa
Poisson’s ratio	ν	0.30
Yield strength	S_y_	308 MPa
Ultimate tensile strength	S_u_	577 MPa
Internal design pressure	p_c_	0.45 MPa

**Table 2:** Stress verification results according to ASME Section VIII Division 2.

SCL	Primary Membrane Stress P_m_ (MPa)	Primary Bending Stress P_b_ (MPa)	Primary Stress Intensity from Linearized (P_m_ + P_b_) Tensor (MPa)	Allowable Limit [1.5f, S_y_] (MPa)	Stress Intensity from Linearized (P_m_ + P_b_ + Q) Tensor (MPa)	Allowable Limit Max [3f, 2S_y_] (MPa)
SCL1 (A1–A2)	33.06	8.70	40.81	308	—	616
SCL2 (B1–B2)	39.52	16.08	56.27	308	—	616
SCL3 (E1–E2)	83.70	36.80	109.50	308	109.50	616
SCL4 (C1–C2)	39.52	20.49	28.85	308	28.85	616

## Data Availability

The original contributions presented in this study are included in the article. Further inquiries can be directed to the corresponding authors.

## Nomenclature

The following abbreviations are used in this manuscript:

Symbol	Description	Unit
(D_i_)	Internal diameter of the cylindrical shell	mm
(R_m_)	Mean radius of the cylindrical shell	mm
(S_M_)	Wall thickness of the cylindrical shell	mm
(S_F_)	Wall thickness of the ellipsoidal head	mm
(H_i_)	Internal height of the ellipsoidal head	mm
(H)	Mean height of the ellipsoidal head	mm
(k_e_)	Ellipticity coefficient of the ellipsoidal head	–
(L)	Characteristic calculation length	mm
(E)	Young’s modulus	MPa
(ν)	Poisson’s ratio	–
(S_y_)	Yield strength of the material	MPa
(S_u_)	Ultimate tensile strength of the material	MPa
(S_a_)	Allowable stress according to ASME Section VIII Division 2	MPa
(p_c_)	Internal design pressure	MPa
(σ)	Total stress	MPa
(σ_p_)	Stress due to internal pressure	MPa
(σ_SUS_)	Stress due to sustained loads	MPa
(σ_EXP_)	Stress due to thermal expansion	MPa
(σ_OCC_)	Stress due to occasional loads	MPa
(σ_m_)	Meridional stress	MPa
(σ_t_)	Circumferential (hoop) stress	MPa
(σ_eq_)	Equivalent stress	MPa
(σ_ech_)	Equivalent stress according to the maximum shear stress criterion	MPa
(σ_memb_)	Membrane stress component	MPa
(σ_bend_)	Bending stress component	MPa
(P_m_)	Primary membrane stress intensity	MPa
(P_b_)	Primary bending stress intensity	MPa
(Q)	Secondary stress intensity	MPa
(N_t_)	Circumferential membrane force per unit length	N/mm
(Mt)	Circumferential bending moment per unit length	(N·mm)/mm
(M_0_)	Bending moment per unit length at the head–shell junction	(N·mm)/mm
(Q_0_)	Transverse shear force per unit length at the junction	N/mm
(Δr)	Radial displacement	mm
(Δr_F_)	Radial displacement of the ellipsoidal head at the junction	mm
(Δr_M_)	Radial displacement of the cylindrical shell at the junction	mm
($\Delta r_{F}^{p}$)	Radial displacement of the head produced by internal pressure	mm
($\Delta r_{M}^{p}$)	Radial displacement of the shell produced by internal pressure	mm
(θ_F_)	Rotation of the ellipsoidal head at the junction	rad
(θ_M_)	Rotation of the cylindrical shell at the junction	rad
($\theta_{F}^{p}$)	Rotation of the head produced by internal pressure	rad
($\theta_{M}^{p}$)	Rotation of the shell produced by internal pressure	rad
(δ)	Displacement influence coefficient	–
(η)	Rotation influence coefficient	–
(φ)	Angular coordinate along the ellipsoidal head meridian	rad
(x)	Meridional coordinate along the cylindrical shell measured from the junction	mm
(F)	Ellipsoidal head component	–
(M)	Cylindrical shell component	–
(+)	Inner surface of the shell	–
(−)	Outer surface of the shell	–
SCL	Stress Classification Line used for stress linearization	–
FEA	Finite Element Analysis	–
ASME	American Society of Mechanical Engineers	–
